# Effects of lipid concentration on thermophilic anaerobic co-digestion of food waste and grease waste in a siphon-driven self-agitated anaerobic reactor

**DOI:** 10.1016/j.btre.2018.e00269

**Published:** 2018-06-26

**Authors:** Yong Hu, Takuro Kobayashi, Guangyin Zhen, Chen Shi, Kai-Qin Xu

**Affiliations:** a Center for Material Cycles and Waste Management Research, National Institute for Environmental Studies, Tsukuba, 305-8506, Japan; b School of Ecological and Environmental Sciences, East China Normal University, Shanghai, 200241, China; c Life and Environmental Sciences, University of Tsukuba, Tsukuba, 305-0005, Japan

**Keywords:** Siphon-driven self-agitated anaerobic reactor (SDSAR), Lipid concentration, Thermophilic, Co-digestion, Methane

## Abstract

To investigate the influence of lipid concentration (of total solids, w/w) on anaerobic treatment of food waste under thermophilic condition, a siphon-driven self-agitated anaerobic reactor was operated for 220 days. The average lipid concentration was changed from 12.8% to 59.3% (w/w) step by step. The gas production rate increased from 1.97 to 2.31 L/L/d with lipid concentration increased from 12.8% to 19.7% (w/w), whereas decreased sharply to 0.78 L/L/d when the concentration further increased to 59.3% (w/w). The COD recovery from output at different lipid concentration was analyzed in this study. With the concentration increased from 12.8% to 59.3% (w/w), the percentage of COD recovered as methane gas decreased from 80.9% to 35.4%, while the percentage of COD remained in the effluent was also decreased significantly from 15.5% to 2.60%. The lipid concentration under 40% (w/w) was recommended in the co-digestion of food waste and grease trap waste.

## Highlights

• Anaerobic co-digestion of grease trap waste and food waste is feasible.
• Higher methane yield was achieved in the co-digestion compared with mono-digestion.
• Biogas production decreased due to lipid accumulation at high lipid concentrations.
• Temperature and mixing frequency play a key role in the lipid distribution.
• Lipid concentration under 40% is recommended in the co-digestion.

## 1 Introduction

High-strength lipid wastes FOG (fat, oil, and grease) normally could not be directly released to the collection system in many metropolitan areas. This is mainly because lipid wastes can easily accumulate in drainage pipes, and forming hardened deposits through a chemical reaction or a physical aggregation process [1]. These deposits lead to a reduction in conveyance capacity and ultimately to sanitary sewer overflow that cost municipalities millions of dollars each year in cleaning, repairing, and maintenance fees [2]. For this reason, grease abatement devices are commonly applied to the kitchen waste streams, and then the collected grease trap waste (GTW) would be most ended up in landfills or incinerators. Alternatively due to its high lipid content, GTW is considered as a suitable source for anaerobic treatment and a potential energy source, since lipids have a significant methane yield (MY) when compared to other organic compounds (such as carbohydrates and proteins) [[3], [4], [5]]. However, there are still distinct disadvantages of lipid-rich waste like GTW when it is used as a sole carbon resource in anaerobic fermentation. The two main disadvantages are operational problems such as clogging, foaming and biomass flotation [6,7], and inhibition problems caused by long-chain fatty acids (LCFA) that exist in lipids [[8], [9], [10], [11]]. On the other hand, anaerobic co-digestion is reported to offer benefits such as increased degradation of organic wastes and dilution of inhibition compounds compared with mono-digestion [19]. Thus, a growing number of researchers have investigated the co-digestion of lipid-rich waste with other organic wastes [[12], [13], [14]].

The biggest advantage of using GTW is that an improved biogas production could be achieved in the anaerobic co-digestion. It is reported that the produced biogas may allow wastewater plants to meet over 50% of their electricity demand through on-site biogas electricity generation [15]. Furthermore, several studies reported the higher MY in co-digestion of GTW and sewage sludge (SS) compared with SS mono-digestion [[16], [17], [18]]. This was likely due to the high biodegradability of GTW (probably close to 100%) compared with that of SS (around 60%) [19]. For the same reason, the co-digestion of GTW and food waste (FW) should also achieve a higher MY compared with the single FW anaerobic digestion. Additionally, since GTW and SS come from different places, collection and transportation of GTW to the wastewater treatment plant could be necessary. On the opposite, it is much easier to conduct an on-site anaerobic co-digestion of GTW and FW due to the close proximity of these two wastes.

In the co-digestion of GTW and SS, it is reported that the maximum MY can be achieved at a grease content of 46% on a VS basis [16], whereas other researchers found an increased MY up to 137% at a grease content on a VS basis [17]. It seems that the lipid content plays an important role in the anaerobic co-digestion process performance, especially in the methane gas production. However, few researchers investigated the effects of lipid content on the co-digestion performance of GTW and FW. Additionally, studies about co-digestion of lipid-rich wastes and other organic wastes often used mesophilic condition as it reduces cost related to heating [20]. Few studies have been conducted in the co-digestion of lipid-rich wastes with SS, and observed an improved biogas production under thermophilic condition [21,22]. In our previous study, the feasibility of using siphon-driven self-agitated anaerobic reactor (SDSAR) for the on-site FW treatment was evaluated, and the SDSAR has been demonstrated that there is better stability under thermophilic condition than mesophilic condition [23]. However, the co-digestion of GTW and FW under thermophilic condition still requires further research especially in determining the optimum lipid concentration in order to reach the maximized methane gas production.

The present study was to assess the effects of lipids originated from restaurant wastewater (in the form of GTW) on the co-digestion performance along with FW under thermophilic condition. A SDSAR was operated continuously and the biogas production, organic removal, and solid removal under different lipid concentrations (of total solids, w/w) were investigated. In addition, the sludge distribution, pH, and the mixing frequency in the SDSAR were also studied. Finally, the scum formed in the reactor was analyzed after day 220.

## 2 Materials and methods

### 2.1 SDSAR system

**Fig. 1:**
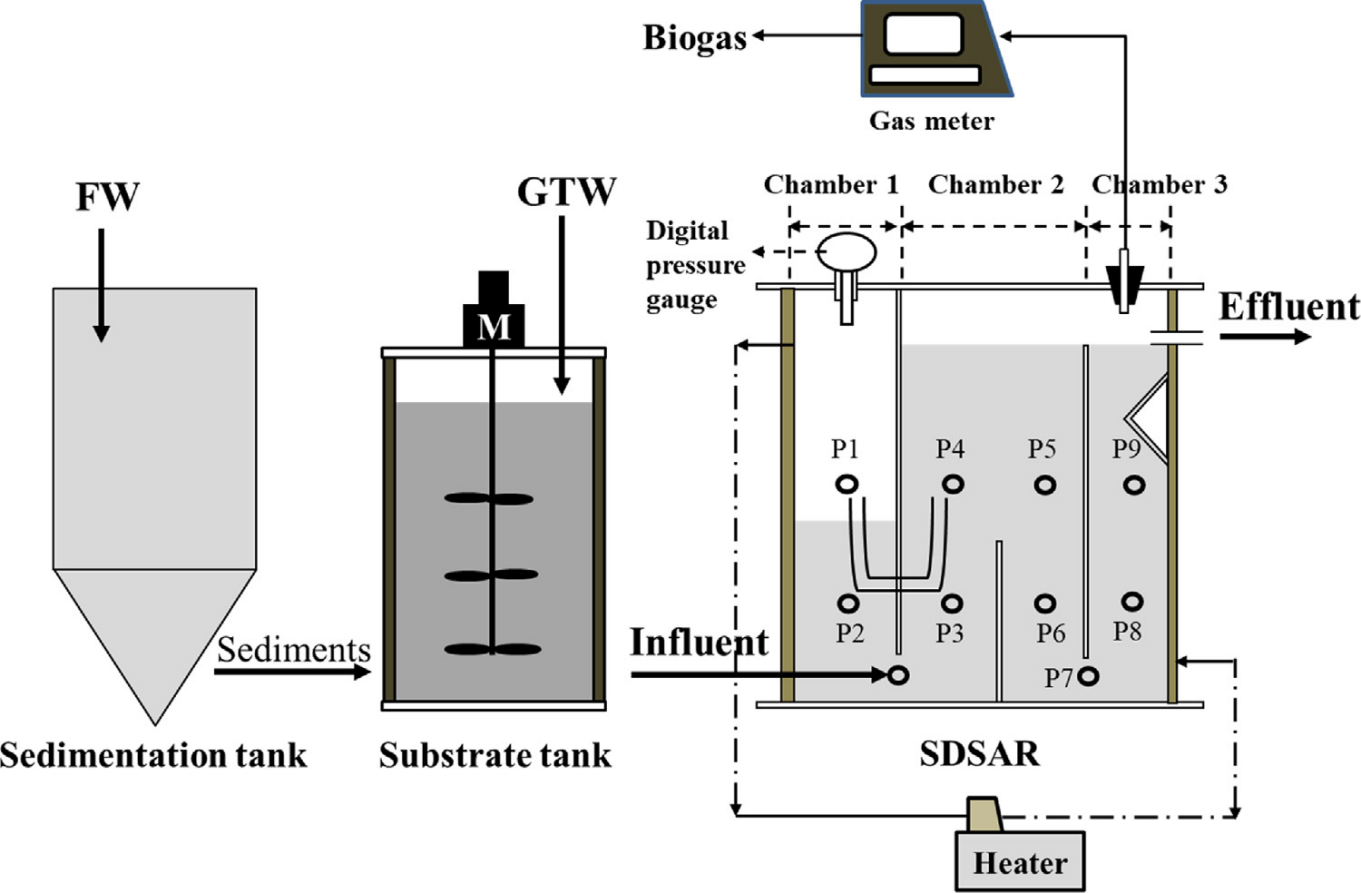
Schematic diagram of the SDSAR system.

A schematic diagram of the SDSAR system applied in this study is shown in Fig. 1. The reactor was made of thermal resistant polyvinyl chloride with an effective volume of 10 L. Beside the influent and effluent ports, there are 9 sampling ports placed on one side of the reactor body. The temperature of the reactor was maintained by water jacket and controlled at 55 ± 1°C. The pressure in the reactor Chamber 1 was recorded by a digital pressure gauge (Krone, KDM30) once per 5 min. The FW and GTW were pumped from the substrate tank to the influent pot of the SDSAR. The substrate tank has an effective volume of 5 L and was kept at a room temperature of around 20°C without cooling.

### 2.2 Feed stock and seed sludge

The raw and cooked FW was collected from the canteen of the National Institute for Environmental Studies, Tsukuba, Japan. The collected FW was first diluted with tap water, and then cut in a disposal machine (Cuisinart, DLC-NXJ2PS). After that, the crushed FW was settled in a settlement tank full with water for 24 h. The assessed water use in this process was kept at around 12 L water to 1 kg FW. Finally, the settled FW was transferred to the substrate tank. GTW was fetched from the grease traps of 11 different restaurants in Japan, mainly supplying meat products and serving for banquets, to make the oily substrate representative. The mixed GTW was heated to 60 °C for at least 6 h to separate the oily layer from the mixture for biodiesel production [24]. After the upper oil layer was pumped away, the prepared GTW residue was stored in a plastic bucket, and was kept at 4 °C prior to use. Before pumped into the SDSAR, the desired proportion of GTW was added into the substrate tank, and mixed completely with the prepared FW by the magnetic stirrer equipped in the tank. To prevent the trace elements deficiencies in the reactors, Fe, Co and Ni were added artificially. The trace elements concentrations in the substrate were as follows: 100 mg-Fe/L, 10 mg-Co/L, and 10 mg-Ni/L, respectively [25]. The reactor was inoculated with sludge cultivated from the SDSAR treating FW under thermophilic condition for more than one year [23].

### 2.3 Operational conditions

The SDSAR was operated for more than 220 days. As shown in Table 1, at the beginning (day 1–15) of this experiment, the reactor was feed with FW only at a HRT of 15 d as start-up stage. After day 16, the HRT was maintained at 10 d, and the organic loading rate (OLR) was set at around 7 gCOD/L/d which was considered to be suitable for the thermophilic FW digestion [23]. Meanwhile, the lipid concentration was increased from 12.8% to 59.3% (w/w) by adjusting the amount of FW and GTW added. When the total volume of FW and GTW in the feed was lower than the total volume of 1 L required to keep HRT constant at 10 d, water was supplemented to the feed. Table 2 shows the characteristics of prepared substrate at different experimental stages.

**Table 1:** Summary of the experimental conditions.

Stage	Time (days)	HRT (d)	lipid concentration (%TS, w/w)	OLR (gCOD/L/d)
1	1–15 (start-up)	15	–	5.75 ± 1.24
2	16–52	10	12.8 ± 1.18	7.26 ± 0.82
3	53–85	10	19.7 ± 0.96	8.22 ± 1.22
4	86–118	10	40.9 ± 2.04	6.15 ± 0.64
5	119–149	10	50.2 ± 2.78	5.92 ± 1.39
6	150–200	10	59.3 ± 3.87	6.43 ± 0.94

**Table 2:** Characteristics of prepared substrates.

lipid concentration (%TS, w/w)	Influent COD (g/L)	Influent pH	Influent TS (g/L)	Influent VS (g/L)	Influent Lipid (g/L)
12.8 ± 1.18	72.6 ± 8.23	3.64 ± 0.33	48.7 ± 5.87	47.3 ± 5.89	6.20 ± 0.49
19.7 ± 0.96	82.2 ± 12.2	3.64 ± 0.30	48.4 ± 5.23	46.8 ± 5.08	9.55 ± 1.53
40.9 ± 2.04	61.5 ± 6.41	3.43 ± 0.23	30.8 ± 6.51	29.6 ± 6.32	13.0 ± 1.38
50.2 ± 2.78	59.2 ± 13.9	3.57 ± 0.33	26.4 ± 1.71	25.3 ± 2.07	14.2 ± 0.63
59.3 ± 3.87	64.3 ± 9.42	4.23 ± 0.81	24.8 ± 2.58	24.1 ± 2.60	16.1 ± 1.08

### 2.4 Analytical methods

The biogas production in the SDSAR was measured with a μFlow gas meter (Bioprocess Control AB). The biogas contents (CH_4_, CO_2_ and N_2_) were determined by a gas chromatography (GC-8 A, Shimadzu). The temperatures of the injector, detector and column were set at 160 °C, 160 °C and 100 °C, respectively. The influents and effluents of the reactor were sampled for chemical oxygen demand (COD), pH, volatile fatty acids (VFA), total solids (TS) and volatile solids (VS) twice a week. Sludge inside the reactor was sampled and analyzed for pH, TS and VS at least once under different lipid concentrations, and average values were calculated. The pH was measured with a pH meter (TOA-DKK), while TS, VS and COD were measured according to the Standard Method [26]. Samples for the analysis of VFA were centrifuged at 15,307*g* for 8 min and filtered with 0.45 μm pore size filters as a pretreatment. The concentrations of VFA were detected by a gas chromatography (GC-2014, Shimadzu). The sample was acidified by adding 0.5 mL of 0.1 mol/L HCl solution to 0.5 mL filtrate, and then 0.1 μL mixed solution was injected to GC for analysis. Lipid content in the substrate and effluent was extracted with a mixture chloroform: methanol 1:2 (v/v), and weighed after dried. In order to evaluate the distribution of lipid in the mixture of sludge, GTW and the cultivated sludge (day 221) was added and mixed completely at a ratio of 25% (v/v%) in a beaker with an effective volume of 100 mL. Then the mixture was heated at 35, 55 and 65 ℃ for 2 h without mixing, respectively.

## 3 Results and discussion

### 3.1 Reactor performance in whole experiment periods

#### 3.1.1 Lipid removal

**Fig. 2:**
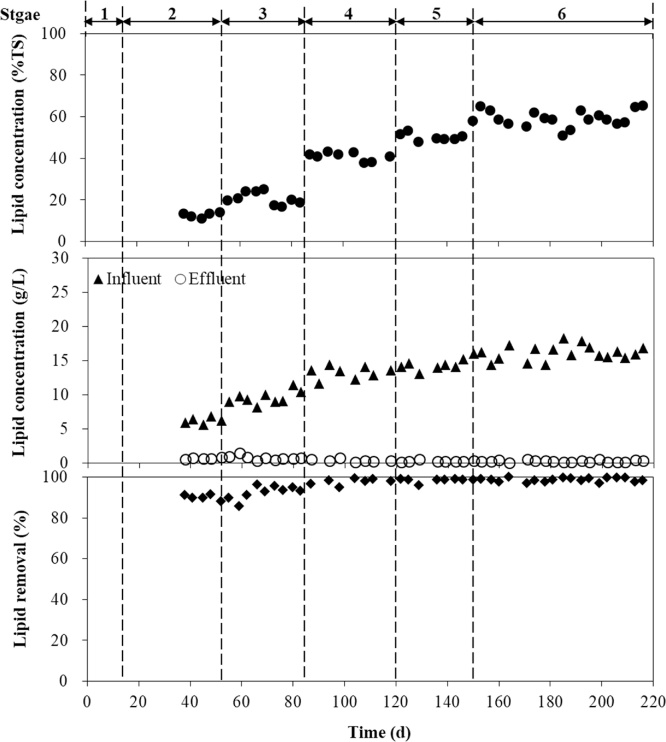
Influent lipid concentration and lipid removal in the SDSAR reactor.

The reactor performance of influent lipid concentration and lipid removal overall experimental period is shown in Fig. 2. At stage 1, lipid from GTW was not added and measured. At a HRT of 10d, without GTW addition (stage 2), the average influent lipid concentration (existed in FW) was 6.2 g/L (12.8% w/w of TS), respectively. After day 53, during the co-digestion of GTW and FW, the influent lipid concentration was increased to 16.1 g/L (59.3% w/w of TS), step by step. When the lipid concentration was under 20% (w/w), the lipid removal was maintained at around 90%. Additionally, with this concentration above 40% (w/w), the lipid removal was higher than 95%, and the effluent lipid concentration was only around 200 mg/L. This concentration is much lower than the reported effluent lipid concentrations (1.1 to 4.5 g/L) in the co-digestion of GTW and FW using a mesophilic semi-continuous anaerobic digester [27].

#### 3.1.2 Biogas production and organic removal

**Fig. 3:**
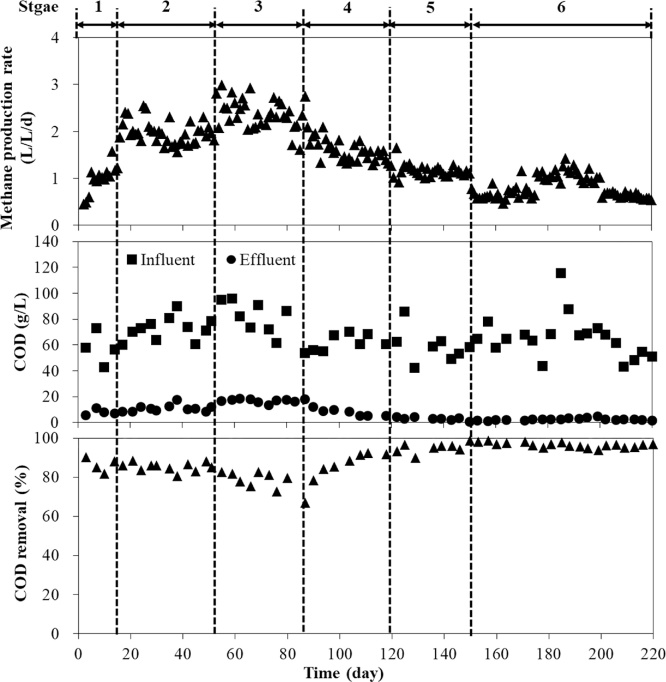
SDSAR reactor performance on biogas production and COD removal.

The methane production and COD removal performance in the SDSAR are shown in Fig. 3. At lipid concentration of 12.8% (mono-digestion of FW), the average methane production rate was 1.97 L/L/d. During the co-digestion, when the concentration was increased to 19.7% (w/w), the methane production rate increased slightly to 2.31 L/L/d. However, when this concentration was further increased to more than 40%, the methane production rate decreased to less than 1.63 L/L/d. With the lipid concentration as high as 59.3% (w/w), the methane production rate was as low as 0.78 L/L/d. The methane content of produced biogas was kept approximately constant at around 63% in this study.

**Fig. 4:**
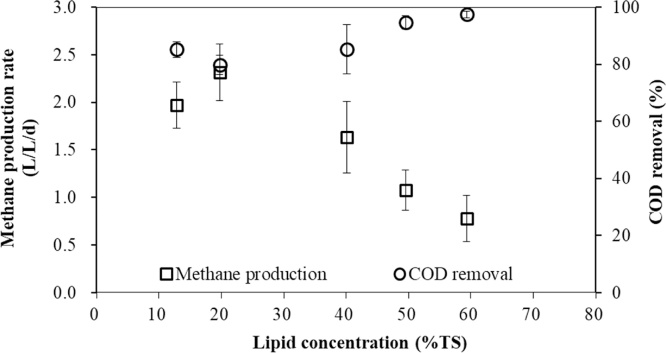
Average methane production rate and COD removal at different lipid concentrations.

According to Fig. 3, The influent COD was not so stable since the composition of FW obtained from the canteen was constantly shifting and changing. In contrast, the effluent COD concentration did change a lot at each lipid concentration condition. When the lipid concentrations were under 40% (w/w), the COD removal was maintained at around 80%. With this concentration increased to 59.3% (w/w), the COD removal increased significantly to around 97%. Especially, the average effluent COD concentration was 1.64 g/L at the lipid concentration of 59.3% (w/w). The average methane production rate and COD removal at different lipid concentrations are shown in Fig. 4.

As summarized in Table 3, the influent TS was around 50 g/L at a relatively low lipid concentration of less than 20% (w/w). When the lipid concentrations were adjusted to more than 20% (w/w), to keep the OLR at around 7 gCOD/L/d with a constant HRT (10 d), water was supplied to the substrate and made the influent TS decreased to around 25 g/L. The average TS removal increased from 83.1% to 91.1% with lipid concentration increased from 12.8% to 59.3% (w/w), while the average VS removal also increased from 86.3% to 93.2%. Under the mesophilic condition, relatively low VS removals (45% to 67%) were reported in the co-digestion of GTW and SS using completely stirred reactors [16,28,29]. A higher VS removal of 77.1% was achieved in the co-digestion of GTW and FW using mesophilic completely stirred reactor [27]. This was probably due to the higher biodegradability of FW compared with SS. In addition, even higher VS removals under the thermophilic (70.6%–76.3%) and hyper-thermophilic (76.8%–86.0%) conditions were obtained in a completely stirred reactor [22]. These results indicated that, the SDSAR has a better solids-handling capacity in the co-digestion of GTW and FW compared with the completely stirring reactor under thermophilic condition. The effluent pH was maintained above 7.5 throughout the experimental periods, and VFA was not detected from the effluent.

**Table 3:** Summary of the reactor performance at different lipid concentrations.

lipid concentration (%TS, w/w)	Effluent pH	TS removal (%)	VS removal (%)	Lipid removal (%)
12.8 ± 1.18	7.84 ± 0.19	83.1 ± 3.79	86.3 ± 3.46	90.0 ± 1.37
19.7 ± 0.96	7.74 ± 0.17	81.9 ± 4.04	84.3 ± 3.57	91.8 ± 3.67
40.9 ± 2.04	7.79 ± 0.15	82.5 ± 4.59	86.9 ± 3.98	97.7 ± 1.54
50.2 ± 2.78	7.82 ± 0.20	87.7 ± 1.24	90.9 ± 1.27	98.3 ± 1.17
59.3 ± 3.87	7.84 ± 0.22	91.1 ± 1.17	93.2 ± 1.08	98.7 ± 0.97

### 3.2 Effects of lipid concentration on SDSAR performance

#### 3.2.1 COD recovery

**Fig. 5:**
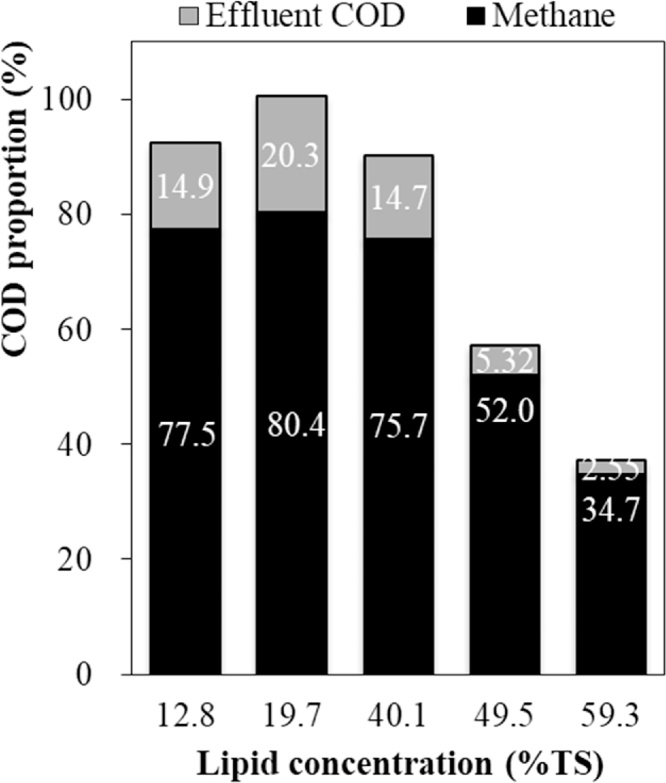
COD recover from output at different lipid concentrations.

The COD recovery as methane at each lipid concentration is shown in Fig. 5. When the lipid concentration was less than 40% (w/w), more than 75% influent COD was converted into methane gas. The rest of influent COD was not converted and remained in the effluent. However, as the lipid concentration increased to 49.5% (w/w), the methane recovery from biogas decreased to 52.0%. The methane recovery further decreased to 34.7% with the concentration increased to 59.3% (w/w). More importantly, as the proportions of effluent COD at these two lipid concentrations were only 5.32% and 2.55% (w/w), large parts of the influent COD should still stay inside the SDSAR.

#### 3.2.2 Methane yield

The average methane yields at various lipid concentrations in different studies are summarized in Table 4. In this study, without GTW addition (lipid concentration of 12.8%, w/w), the MY from FW was 416 mLCH_4_/gVS, which was much higher than the reported value of 264 mLCH_4_/gVS using a continuous stirred tank reactor (CSTR) treating FW under thermophilic condition [30], but lower than 462 mLCH_4_/gVS in our previous study using the same reactor at HRT of 10d [23]. Theoretical CH_4_ productions of carbohydrate, protein and lipid were 415, 496 and 1014 mLCH_4_/gVS [31]. Thus, the increased MY can be achieved with increasing the influent lipid concentration, theoretically. However, in the co-digestion of FW and GTW in this study, at lipid concentrations of 19.7%, 40.9% and 50.2% (w/w), the MYs were 493, 550 and 427 mLCH_4_/gVS. Compared with mono-digestion of FW, the MYs in co-digestion increased 18.5%, 32.2% and 2.64%, respectively. In contrast, the MY decreased to 323 mLCH_4_/gVS when the concentration increased to 59.3% (w/w). In the previous studies, the MYs increased with the lipid concentration increasing till its peak value. On the other hand, the further increasing in lipid concentration led to an inhibited MY [16,32], or even caused process failure [17]. Researchers attributed this to VFA accumulation which causes acidification in the digester, or the relatively short HRT which lead to the washout of microorganisms during removal of effluent [16,32]. However, acidification and microorganisms washout was not observed in the effluent in this study.

**Table 4:** Comparison of methane yield at various lipid concentrations in different studies.

Study	Substrate	T (°C)	Mixing model	lipid concentration (%TS, w/w)	lipid concentration (%VS, w/w)	Methane yield (ml-CH_4_/g-VS)	OLR (gVS/L/d)
This study	FW + GTW	55	siphon-mixing	12.8	13.1	416	4.73 ± 0.59
				19.7	20.4	493	4.68 ± 0.51
				40.9	43.9	550	2.96 ± 0.63
				50.2	56.1	427	2.53 ± 0.21
				59.3	66.8	323	2.41 ± 0.26

Reference [32]	MBW + WAS	35	constant mixing (15 min/2 h)		18	420	1.5
					25	446	2.5
					40	515	4.0
					55	625	6.0
					60	706	8.0
					65	35	10.0

Reference [16]	SS + GTW	35	constant mixing (300 rpm)		20	441	1.93–2.45
					28	444	2.8
					38	447	3.13
					46	463	3.46
					55	318	3.99
					71	315	4.41

Reference [17]	WAS + FOG	37	constant mixing (1000 rpm)		0	252	2.34
					64	598	2.34
					75	252	3.4

(MBW: municipal biomass waste; WAS: waste-activated sludge).

#### 3.2.3 Sludge distribution, mixing frequency, and scum accumulation

**Fig. 6:**
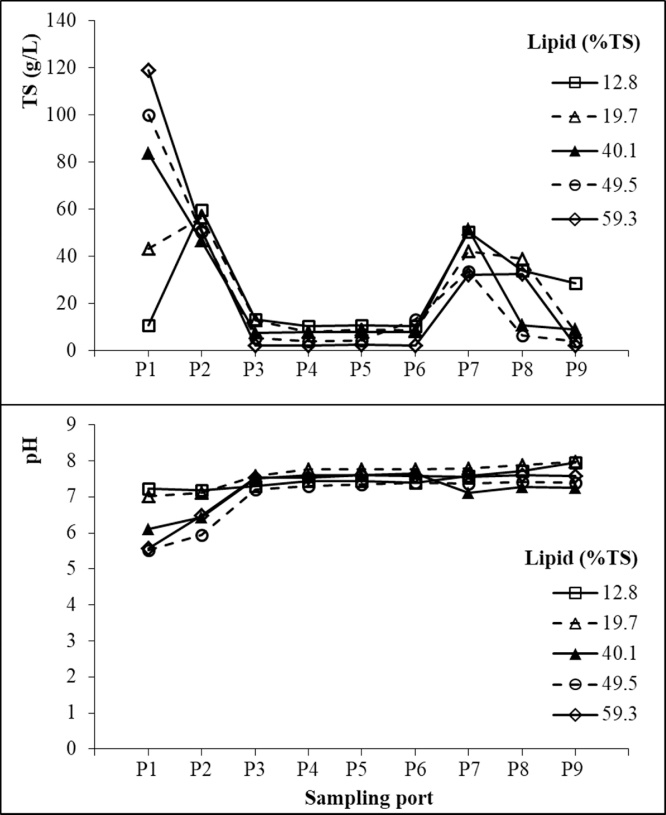
Distribution of TS and pH in the SDSAR reactor at different lipid concentrations.

The average TS and pH in the SDSAR at different lipid concentrations are shown in Fig. 6. Sludge inside the reactor was taken from sampling port P1–P9 as shown in Fig. 1. There is no significant difference between TS and VS distribution in the SDSAR. In Chamber 1, at a lipid concentration of 12.8% (w/w), the TS and VS concentrations in P1 (10.5 and 8.72 g/L) were much lower than those from P2 (59.6 and 55.3 g/L) since the position of P2 was much lower when compared with P1. However, with the lipid concentration increased to 59.3% (w/w), the TS and VS in P1 increased sharply to 119 and 117 g/L while those in P2 did not change that much. As a result, the TS and VS concentrations in P1 became almost 2 times more than those in P2. According to the lipid analysis of the sludge samples taken from P1 which were completed on day 181 and 189, over 95% of the samples were composed of lipid. These results suggest that lipid accumulated in Chamber 1 and formed the scum layer in the reactor. The lipid accumulation could be contributed to the lower degradation rate of lipid in comparison to e.g. sugar or protein [32]. Therefore, with its concentration increases, lipid can easily be accumulated in the anaerobic digesters [33]. In addition, the accumulated lipid caused a low pH of 5.6 in Chamber 1 at a lipid concentration of 59.3% (w/w).

In Chamber 2, no big difference was observed between P3 to P6 at each lipid concentration. However, the average TS decreased significantly from around 10 g/L at a lipid concentration of 12.8% (w/w) to only around 2 g/L at the concentration of 59.3% (w/w). A similar TS tendency with lipid concentration changing was observed in Chamber 3. Additionally, the TS concentration in Chamber 3 was higher than that in Chamber 2. This result could be explained as that Chamber 2 would function most likely a CSTR whereas Chamber 3 would function like an unmixed plug flow reactor according to their fluid analysis [34]. On day 221, the reactor was opened from its upper side, and a scum layer over 10 cm thick was found inside the reactor. Unlike the scum layer formed in Chamber 1 which was almost made of lipid, the scum layer formed in Chambers 2 and 3 had a relatively high solid content (TS 26.4%). The scum TS value is even much higher than the average sludge TS concentration in Chamber 2. This result indicated that the accumulated lipid in the reactor brought sludge to the upper side in Chambers 2 and 3, and finally formed a thick scum layer. However, throughout the experimental periods, block problem did not occur in this SDSAR. In Chambers 2 and 3, pH was kept above 7 overall the experimental periods even when the scum layer appeared.

**Fig. 7:**
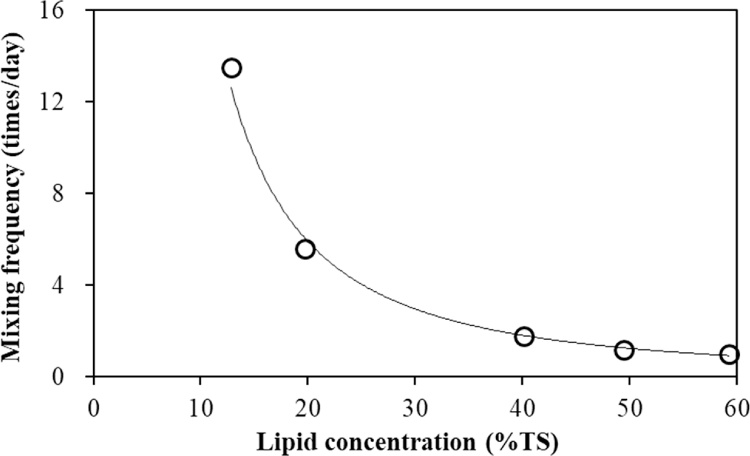
Distribution of lipid in the mixture of sludge and lipid at different temperatures.

In this study, the pressure variation of more than 0.5 atm was considered as mixing completed once in the reactor. Depending on the data recorded by the digital pressure gauge, the average mixing frequencies at different lipid concentrations are calculated and are shown in Fig. 7. The average siphon mixing frequency at a lipid concentration of 12.8% (w/w) was 13.5 times per day. However, when this concentration increased to 19.7% (w/w), the mixing frequency decreased sharply to 5.6 times per day. At lipid concentrations higher than 40% (w/w), the mixing frequencies further decrease to around 1–2 times per day. According to these results, the low mixing frequencies did not significant affect the methane production under low lipid concentrations. In contrast, the highest MY was achieved at a mixing frequency of only 1.8 times per day. Previous studies stated that the abundance of the methanogenesis showed a significant increase at low mixing frequency (50–60 rpm) or minimal rather than high [35]. On the other hand, the SDSAR with siphon-mixed model had higher MY compared with other anaerobic digesters with unmixed or continuously mixed models in the FW treatment [36]. However, the experimental results in present study showed no significant correlations between MY and siphon-mixing frequency at various lipid concentrations. The low MY and low mixing frequency were most probably caused by accumulated lipid and scum layer in the SDSAR. In other word, lipid concentration played the key role in the co-digestion of FW and GTW.

The effects of temperature on the distribution of lipid in the mixture of sludge and lipid were evaluated, and the results are shown in Fig. S1 (Supplementary material). Only small amount of lipid floated up after heating at 35°C for 2 h. In contrast to this, approximately 40% of the added lipid floated at the top of the mixture after heating at 55°C for 2 h, and the value increased to 88% after heating at 65°C for 2 h. These results demonstrated that GTW can easily float up and form a lipid layer under thermophilic and hyper-thermophilic conditions. Therefore, sufficient mixing which can help lipid dispersed in the sludge mixture becomes much more important in the anaerobic lipid-rich waste digestion. Moreover, this could explain why there was no lipid accumulated in the mesophilic anaerobic digesters even at high lipid concentration (of VS, w/w) more than 60% [17,32], and also why the MY decreased sharply without acidification and biomass washout in the effluent in our study. Consequently, to avoid lipid accumulation, the lipid concentration of less than 40% (w/w) is recommended in the SDSAR reactor under thermophilic condition.

## 4 Conclusions

The effects of lipid concentration on the thermophilic co-digestion performance of FW and GTW in a SDSAR were evaluated. The highest methane yield of 550 mLCH_4_/gTS was achieved at a lipid concentration of 40.9% (w/w). Then, the methane yield decreased sharply to 323 mLCH_4_/gTS when this concentration further increased to 59.3% (w/w). The decrease of methane yield was mainly related to lipid accumulation in the reactor. Our results suggest that the SDSAR has better performance in COD and solid removal in the co-digestion of FW and GTW compared with other anaerobic digesters. In addition, to optimize biogas production and maintain a stable process, the lipid concentration under 40% (w/w) is recommended in the co-digestion of FW and GTW using SDSAR under thermophilic condition.

## Declarations of interest

None

## Conflict of interest

All authors declare no actual or potential conflict of interest.
