# Supplementary material for: Effects of lipid concentration on thermophilic anaerobic co-digestion of food waste and grease waste in a siphon-driven self-agitated anaerobic reactor
Source: Biotechnol Rep (Amst). 2018 Jun 26;19:e00269. doi: 10.1016/j.btre.2018.e00269 (PMC6036866; doi:10.1016/j.btre.2018.e00269)
Supplement: Supplementary file 1 [file mmc1.docx]

**Supplementary material**


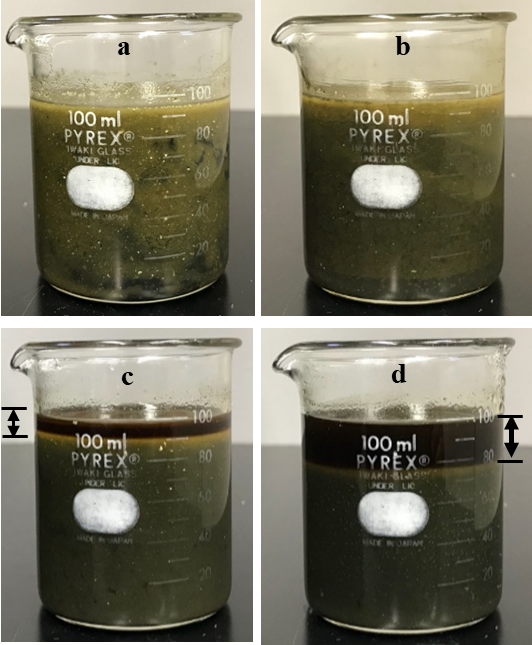


**Fig. S1** Effect of lipid concentration on the mixing frequency in the SDSAR reactor.

(a: mixture under 20 ℃; b: mixture after heating at 35 ℃ for 2h; c: mixture after heating at 55 ℃ for 2h; d: mixture after heating at 65 ℃ for 2h)
